# Supplementary material for: SARS-CoV-2 Neutralizing Antibodies Kinetics Postvaccination in Cancer Patients under Treatment with Immune Checkpoint Inhibition
Source: Cancers (Basel). 2022 Jun 4;14(11):2796. doi: 10.3390/cancers14112796 (PMC9179468; doi:10.3390/cancers14112796)
Supplement: Supplementary file 1 [file cancers-14-02796-s001.zip › cancers-1678535-supplementary.pdf]

Supplementary Files

SARS-CoV-2 Neutralizing Antibodies Kinetics Postvaccination in Cancer Patients under Treatment with Immune Checkpoint Inhibition

Evangelos Terpos, Michalis Liontos, Oraianthi Fiste, Flora Zagouri, Alexandros Briasoulis, Aimilia D. Sklirou, Christos Markellos, Efthymia Skafida, Alkistis Papatheodoridi, Angeliki Andrikopoulou, Konstantinos Koutsoukos, Maria Kaparelou, Vassiliki A. Iconomidou, Ioannis P. Trougakos and Meletios-Athanasios Dimopoulos

Table S1. Case-control comparison regarding baseline characteristics.

Variables	Patient cohort (*n* = 85); Median (IQR)	Control cohort (*n* = 75); Median (IQR)	*p*-Value
**Age**	68.04 (62-77)	65.51 (62-68)	0.12
**BMI**	26.89 (24-29)	26.88 (24-29)	0.47
**Sex**			
Male	52 (61.18%)	31 (41.33%)	0.012
Female	33 (38.82%)	44 (58.67%)	
**Type of vaccine**			
BNT162b2/mRNA1273	68 (80%)	56 (74.67%)	0.45
AZD1222	17 (20%)	19 (25.33%)	
